# Metabolic dysfunction-associated fatty liver disease and implications for cardiovascular risk and disease prevention

**DOI:** 10.1186/s12933-022-01697-0

**Published:** 2022-12-03

**Authors:** Xiao-Dong Zhou, Jingjing Cai, Giovanni Targher, Christopher D. Byrne, Michael D. Shapiro, Ki-Chul Sung, Virend K. Somers, C. Anwar A. Chahal, Jacob George, Li-Li Chen, Yong Zhou, Ming-Hua Zheng, CHESS-MAFLD consortium

**Affiliations:** 1 Department of Cardiovascular Medicine, the Heart Center, the First Affiliated Hospital of Wenzhou Medical University, Wenzhou, China; 2 Department of Cardiology, the Third Xiangya Hospital, Central South University, Changsha, China; 3 Department of Medicine, Section of Endocrinology, Diabetes, and Metabolism, University of Verona, Verona, Italy; 4 Southampton National Institute for Health and Care Research Biomedical Research Centre, University Hospital Southampton, Southampton General Hospital, Southampton, UK; 5 Center for Prevention of Cardiovascular Disease, Section On Cardiovascular Medicine, Wake Forest University School of Medicine, Winston-Salem, NC USA; 6 Department of Internal Medicine, Division of Cardiology, Kangbuk Samsung Hospital, Sungkyunkwan University School of Medicine, Seoul, Korea; 7 Department of Cardiovascular Medicine, Mayo Clinic College of Medicine, Rochester, USA; 8 Division of Cardiovascular Medicine, Hospital of the University of Pennsylvania, Philadelphia, PA USA; 9 Storr Liver Centre, Westmead Institute for Medical Research, Westmead Hospital and University of Sydney, Sydney, NSW Australia; 10 MAFLD Research Center, Department of Hepatology, the First Affiliated Hospital of Wenzhou Medical University, Wenzhou, China; 11 Clinical Research Institute, Shanghai General Hospital, Shanghai Jiaotong University School of Medicine, Shanghai, China; 12 Institute of Hepatology, Wenzhou Medical University, Wenzhou, China; 13 Key Laboratory of Diagnosis and Treatment for the Development of Chronic Liver Disease in Zhejiang Province, Wenzhou, China

**Keywords:** Cardiovascular disease, Metabolic dysfunction-associated fatty liver disease (MAFLD), Non-alcoholic fatty liver disease (NAFLD), Risk factors, Pharmacotherapies

## Abstract

The newly proposed term “metabolic dysfunction-associated fatty liver disease” (MAFLD) is replacing the old term “non-alcoholic fatty liver disease” (NAFLD) in many global regions, because it better reflects the pathophysiology and cardiometabolic implications of this common liver disease. The proposed change in terminology from NAFLD to MAFLD is not simply a single-letter change in an acronym, since MAFLD is defined by a set of specific and positive diagnostic criteria. In particular, the MAFLD definition specifically incorporates within the classification recognized cardiovascular risk factors. Although convincing evidence supports a significant association between both NAFLD and MAFLD, with increased risk of CVD morbidity and mortality, neither NAFLD nor MAFLD have received sufficient attention from the Cardiology community. In fact, there is a paucity of scientific guidelines focusing on this common and burdensome liver disease from cardiovascular professional societies. This Perspective article discusses the rationale and clinical relevance for Cardiologists of the newly proposed MAFLD definition.

## Introduction

Many Cardiologists are not aware of the increased risk of cardiovascular disease (CVD) among patients with non-alcoholic fatty liver disease (NAFLD) [1]. Whilst Cardiologists pay close attention to traditional CVD risk factors, there is currently little awareness that fatty liver per se may contribute to CVD risk. To date, however, it remains debatable whether screening for fatty liver disease should be given the same priority as other established cardiometabolic risk factors. Although NAFLD is associated with increased CVD risk, routine screening is not recommended in current cardiovascular guidelines. It is reasonable to assume that the lack of clear recommendations for NAFLD screening likely relates to the lack of any effective pharmacotherapies other than lifestyle modification. The lack of awareness of the existing link between NAFLD and increased CVD risk further exacerbates clinical inertia amongst Cardiologists, Primary-care practitioners and non-liver clinician specialists [2, 3].

**Fig. 1:**
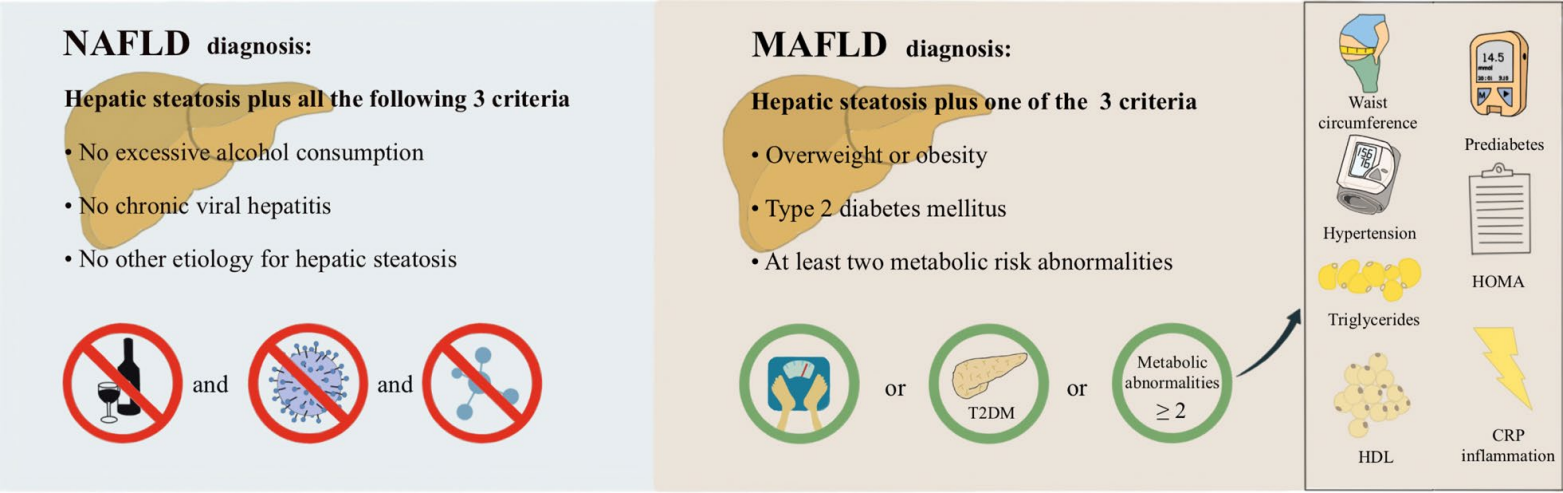
Comparison of diagnostic criteria between NAFLD and MAFLD definitions. Hepatic steatosis is detected either by imaging techniques, blood biomarkers and scores or by liver histology. The definition of NAFLD is based on the evidence of hepatic steatosis *in the absence* of excessive alcohol consumption, chronic viral hepatitis, or other competing causes of hepatic steatosis. The definition of MAFLD is based on the evidence of hepatic steatosis *in the presence* of at least one of the following three metabolic conditions, overweight/obesity, type 2 diabetes, or the presence of at least two of the following metabolic abnormalities: (1) waist circumference ≥ 102/88 cm in Caucasian men and women (or ≥ 90/80 cm in Asian men and women); (2) blood pressure ≥ 130/85 mmHg or specific drug treatment; (3) plasma triglycerides ≥ 150 mg/dl (≥ 1.70 mmol/L) or specific drug treatment; (4) plasma high-density lipoprotein (HDL)-cholesterol < 40 mg/dl (< 1.0 mmol/L) for men and < 50 mg/dl (< 1.3 mmol/L) for women or specific drug treatment; (5) prediabetes (i.e., fasting glucose levels 100 to 125 mg/dl [5.6 to 6.9 mmol/L], or 2-h post-load glucose levels 140 to 199 mg/dl [7.8 to 11.0 mmol] or HbA1c 5.7% to 6.4% [39 to 47 mmol/mol]); (6) Homeostasis model assessment (HOMA) of insulin resistance score ≥ 2.5; and (7) plasma high-sensitivity C-reactive protein (CRP) level > 2 mg/L. Thus, MAFLD diagnosis does not require exclusion of other liver diseases but as a prerequisite it must have evidence of metabolic dysregulation. *NAFLD* non-alcoholic fatty liver disease, *MAFLD* metabolic dysfunction-associated fatty liver disease

In 2020, metabolic dysfunction-associated fatty liver disease (MAFLD) was proposed as a more appropriate term than NAFLD, because this nomenclature better defines the pathophysiology of this liver disease and its associated metabolic abnormalities [4, 5]. The proposed change is more than a name change because it affects how clinicians perceive the association of this common liver disease with CVD and metabolic risk. NAFLD is defined as a group of heterogeneous conditions in which there is liver fat accumulation in the absence of secondary causes of hepatic steatosis, such as excessive alcohol consumption, viral hepatitis and other known causes of hepatic steatosis [6]. These “negative” (by exclusion) diagnostic criteria are not appropriate, meaning that NAFLD is only present when all other causes of fatty liver are excluded. In addition, fatty liver disease may coexist with viral hepatitis, excessive alcohol intake or other liver diseases. This renders it difficult for clinicians to make a definitive diagnosis of NAFLD in the face of other potential causes of hepatic steatosis. The term “non-alcoholic” may also confuse patients in terms of the real cause of their disease, which is not conducive to a good therapeutic relationship. Significantly different from NAFLD, MAFLD is defined as a condition characterized by liver fat accumulation in the presence of at least one of the following three metabolic conditions: overweight/obesity, T2DM, or at least two of seven metabolic risk abnormalities in those subjects who do not have T2DM and are lean by ethnic-specific body mass index (BMI) criteria (Fig. 1) [7]. The “positive” diagnostic criteria for MAFLD are based on the coexistence of hepatic steatosis and metabolic dysfunction and hence MAFLD may also coexist with other liver diseases. This is not possible when using the NAFLD definition, which requires the exclusion of all other causes of hepatic steatosis as a prerequisite for diagnosis. To date, the newly proposed definition of MAFLD has been accepted by many experts in the field, and by some pan-national societies; although debate is ongoing and there is not uniform agreement [8, 9]. For some experts the change in terminology/definition from NAFLD to MAFLD seems premature and they suggest that such a change could also lead to confusion [10, 11]. In addition, there is not consensus on what constitutes “metabolic health”. That said, taken together, the MAFLD definition better emphasizes the pathogenic role of metabolic dysregulation in the development and progression of this common and burdensome liver disease. Additionally, the inclusion of recognized cardiovascular risk factors within the definition, highlights the need for treatment of these specific coexisting cardiometabolic risk factors.

**Fig. 2:**
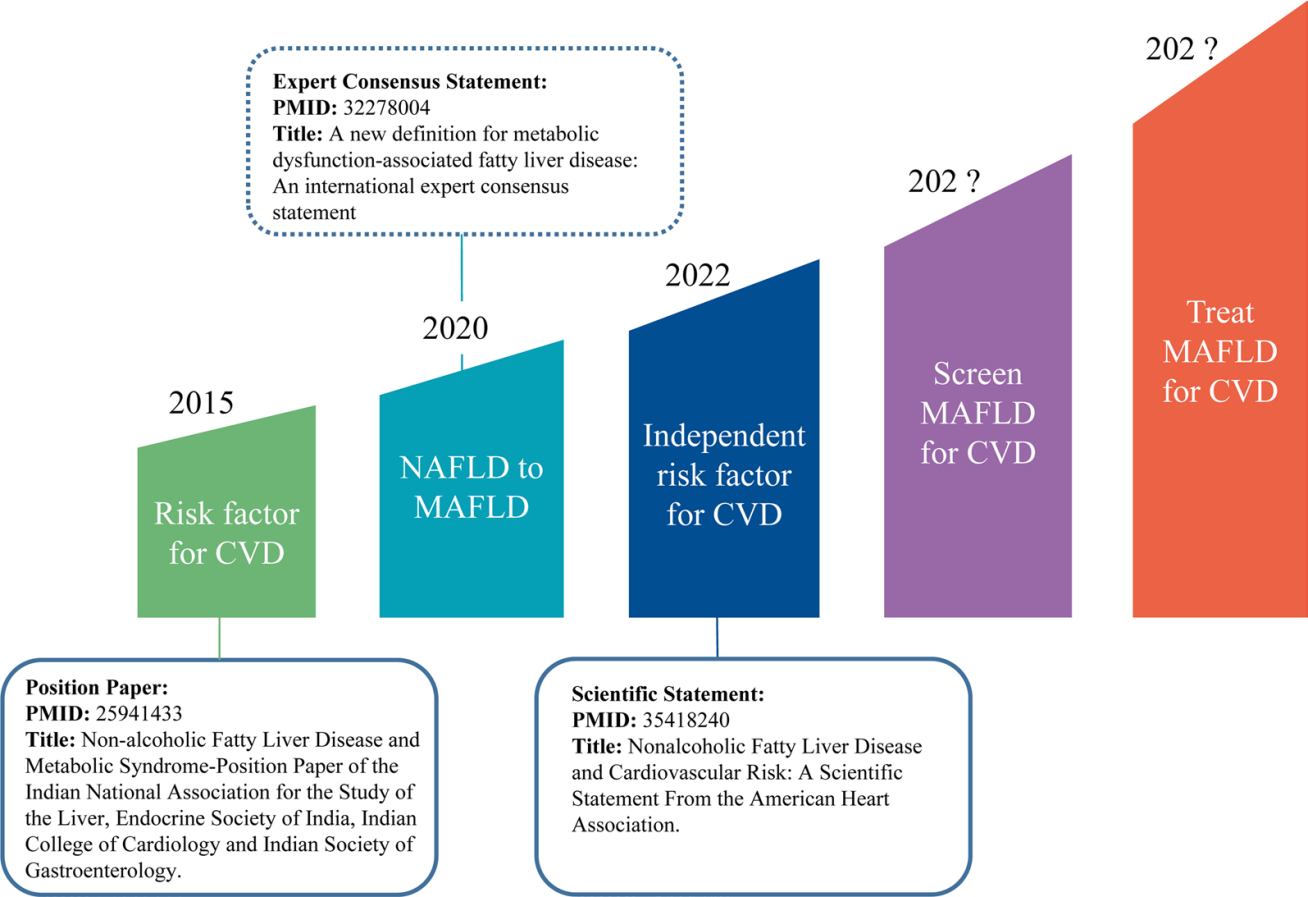
Timescale of the recognition of metabolic dysfunction-associated fatty liver disease (MAFLD) amongst cardiovascular societies

To date, there are few consensus statements about NAFLD or MAFLD published by national or international cardiovascular societies (Fig. 2). The first position paper was published by the Indian College of Cardiology in 2015 and raised questions as to whether NAFLD itself may predispose to CVD risk, independent of other common CVD risk factors [12]. In 2022, the American Heart Association (AHA) issued the first scientific statement on NAFLD and CVD risk [13]. This AHA statement highlighted the strong and independent association between NAFLD and increased risk of CVD and sounded the alarm to increase awareness among clinicians, particularly Cardiologists. We are now at the stage where it is germane to consider and understand the emerging relationship between MAFLD and CVD risk from a Cardiologist’s perspective (Table 1). This Perspectives article discusses issues related to NAFLD and MAFLD that are of concern for Cardiologists, divided into the following five sections: is the estimated risk of CVD similar when using the NAFLD or MAFLD definitions? Why is MAFLD associated with an increased risk of CVD? What is the role of MAFLD in CVD; is it a bystander or a mediator of CVD? Is routine screening for MAFLD necessary for CVD risk assessment? What is the effect of treatment interventions for MAFLD on the risk of CVD?

**Table 1:** Principal epidemiological studies examining the association between MAFLD and the risk of adverse CVD outcomes

Author, Year	Country	MAFLD diagnosis	CVD outcomes	Study population	Study design	Follow-up length	Main findings	Refs.
Liu HH et al. 2021	China	US	MACE defined as CVD death, nonfatal myocardial infarction or coronary revascularizations	3306 patients with CCS with MAFLD; 3306 age- and sex-matched controls without MAFLD	Matched case–control study	Mean of 4.6 years	CCS patients with MAFLD overlapping with NAFLD or MAFLD-only, had a 1.3-fold and 2.3-fold higher risk of MACE compared with controls (both p < 0.05)	[22]
Tsutsumi T et al. 2021	Japan	US	10-year risk ASCVD either by Framingham risk score or by Suita score	2306 subjects with fatty liver with health check-up programs	Cohort study	About 10 years	Cumulative incidence of worsening of the Suita score was higher in the MAFLD group than in the NAFLD group. MAFLD, but not NAFLD, was independently associated with higher 10-year ASCVD risk score	[20]
Kim D et al. 2021	USA	US	All-cause mortality CVD mortality	7761 subjects in the NHANES III 1988–94 database	Population-based cohort study	Median of 23 years	Individuals with MAFLD had a 17% higher risk of all-cause mortality (HR 1.17; 95% CI 1.04–1.32). MAFLD was associated with a higher risk of CVD mortality. NAFLD did not increase the risk of all-cause mortality	[30]
Nguyen VH et al. 2021	USA	US	All-cause mortality CVD mortality	2997 subjects with MAFLD and/or NAFLD in the NHANES III 1988–1994 database	Population-based cohort study	Median of 23 years	MAFLD-only status was independently associated with all-cause mortality compared with NAFLD-only status (adjusted HR 2.4; 95% CI, 1.2–4.6)	[29]
Liu S et al. 2021	China	US	Subclinical atherosclerosis markers (defined as increased ba-PWV increased CIMT, or microalbuminuria)	6232 individuals aged 40 years or older	Population-based cohort study	Median of 4.3 years	MAFLD was associated with higher risks of developing subclinical atherosclerosis. Resolution of MAFLD was associated with lower risks of both increased CIMT and ba-PWV	[21]
Niriella MA et al. 2021	Sri Lanka	US	Fatal and nonfatal CVD events	2985 individuals	Population-based cohort study	Follow-up of 7 years	Subjects excluded by the NAFLD definition but captured by the MAFLD definition had substantially higher risk of adverse CVD outcomes than controls	[28]
Liang Y et al. 2022	China	US	Nonfatal CVD events (coronary heart disease and stroke)	6873 middle-aged individuals	Cohort study	Mean of 4.6 years	MAFLD was associated with higher risk of CVD events (HR 1.44; 95% CI, 1.15–1.81). Similar associations were observed for NAFLD	[120]
Kim H et al. 2022	Korea	US	10-year ASCVD risk by 2018 AHA guideline and coronary artery disease by CCTA	2144 asymptomatic subjects without a prior CVD history with health check-ups	Cross-sectional	None	MAFLD predicted a higher 10-yr ASVD risk and the risk of CCTA-defined coronary artery disease better than NAFLD. NAFLD-only status did not show any association with the 10-year ASCVD risk	[18]
Lee H et al. 2021	Korea	ICD-10 codes	Composite CVD outcome, inclusive of myocardial infarction, stroke, heart failure or CVD mortality	~ 9.5 million subjects undergoing routine NHIS health examinations	Nationwide health screening database	Median of 10.1 years	Change from NAFLD to MAFLD criteria identified a greater number of individuals at risk for CVD events	[14]
Yoneda M et al. 2021	Japan	FLI	CVD events (stroke and coronary artery disease)	~ 4.0 million persons from the Japan Medical Data Center database	Nationwide claims database	2013–2019	Rates of CVD events increased similarly with NAFLD and MAFLD definitions	[52]
Jeong S et al. 2021	Korean	FLI	CVD events (≥ 2 days of hospitalization due to coronary heart disease)	333,389 subjects from Korean NHIS database	Nationwide health screening database	Follow-up of 1850,704 person-year	Coexistence of hepatic steatosis and metabolic dysfunction better predicted CVD events than hepatic steatosis or metabolic dysfunction alone	[121]
Matsubayashi Y et al. 2022	Japan	FLI	CVD events	570,426 subjects from a nationwide claims database	Nationwide claims database	Median of 5.2 years	Differentiating metabolic syndrome and/or MAFLD by gender with or without coexisting type 2 diabetes can help accurately identify patients at high CVD risk	[122]
Noda T et al. 2022	Japan	FLI	All-cause mortality and CVD re-hospitalization events	479 patients with ACS	Retrospective cohort study	Median of 1.4 years	Coexistence of MAFLD and reduced physical function tests independently predicted the risk of clinical outcomes	[23]
Moon JH et al. 2022	Korea	FLI	All-cause mortality and CVD events	8919 subjects from the Ansung-Ansan cohort study	Population-based cohort study	Median of 15.7 years	MAFLD predicted the risk of all-cause mortality and CVD events better than NAFLD. Metabolic dysfunction contributed to all-cause mortality (HR 1.51; 95% CI, 1.21 to 1.89) and CVD events (HR 1.27; 95% CI,1.02 to 1.59)	[50]

*ASCVD*: atherosclerotic cardiovascular disease, *ACS* acute coronary syndrome, *CCS* chronic coronary syndrome, *CCTA* coronary computed tomography angiography, *CIMT* carotid intima-media thickness, *CVD* cardiovascular disease, *FLI* fatty liver index, *ICD* international classification of diseases, *MACE* major adverse cardiac events, *MAFLD* metabolic-associated fatty liver disease, *NAFLD* non-alcoholic fatty liver disease, *NHANES* National Health and Nutrition Examination survey, *NHIS* National Health Insurance service, *US* ultrasonography

## Is the estimated risk of CVD similar when using the NAFLD or MAFLD definitions?

Because the overlap between the NAFLD and MAFLD definitions in the general population is reported to be around 70–90%, it is expected that patients with MAFLD have essentially similar CVD risk to those with NAFLD [14–16]. However, emerging evidence suggests a greater risk of CVD events in patients with MAFLD than in those with NAFLD. Using the National Health and Nutrition Examination Survey (NHANES 1999–2016) database, Zhang et al. [17] reported that patients with MAFLD had a significantly higher 10-year CVD risk (as assessed by the Framingham risk score) compared to those with NAFLD. These data provided the first hint that the CVD risk burden may be greater for MAFLD. Kim et al. [18] analyzed data in 2144 subjects without a prior history of CVD and showed that individuals with MAFLD had a remarkably higher risk of intermediate to high 10-year CVD risk compared to those with NAFLD only (defined as presence of NAFLD but not MAFLD), with an odds ratio (OR) of 8.17 (95% CI 2.40–36.1) in adjusted regression analyses. It is known that the Suita score is a CVD risk prediction tool that may improve CVD risk prediction relative to the Framingham risk score in Japanese individuals [19]. Tsutsumi et al. [20] reported that MAFLD better identified patients at high CVD risk (as estimated by Suita and Framingham risk scores) compared with NAFLD. In a community-based cohort of 6232 participants followed for a median of 4.3 years, Liu et al. [21] reported that MAFLD was associated with a greater risk of developing subclinical atherosclerosis, defined as increased carotid intima-media thickness and plaque, elevated brachial ankle pulse wave velocity, or microalbuminuria. Liu H et al. [22] reported that MAFLD was associated with an increased CVD risk in a cohort of 3306 patients with chronic coronary syndrome. Finally, in a prospective study of nearly 500 hospitalized patients with acute coronary syndromes (ACS) and hepatic steatosis, Noda et al. [23] showed that the coexistence of MAFLD and impaired physical function tests independently predicted the risk of adverse CVD outcomes. Collectively, therefore, accumulating evidence indicates that MAFLD may increase the risk of developing adverse CVD outcomes.

A recent large meta-analysis of 17 observational studies (including more than 12 million individuals) also reported that MAFLD is significantly associated with higher risk of overall mortality (hazard ratio (HR) 1.24, 95% confidence interval [CI] 1.13–1.34), CVD mortality (HR 1.28, 95% CI 1.03–1.53), nonfatal CVD events (HR 1.49, 95% CI 1.34–1.64) and stroke (HR: 1.55, 95% CI 1.37–1.73) [24]. Moreover, a matched cohort study, using electronic primary healthcare databases from four European countries, reported that NAFLD appears not to be significantly associated with risk of acute myocardial infarction or stroke after adjustment for common CVD risk factors, (although it should be noted that in this large registry-based study it was not possible to prove that control subjects did not have NAFLD, giving rise to the potential for misclassification bias attenuating the strength of any association between NAFLD and CVD, towards the null) [25]. Additionally, although there are important limitations of Mendelian randomization studies, a recent study did not find evidence supporting the existence of causal associations of NAFLD *itself* with acute myocardial infarction and any stroke subtypes [26]. In contrast to the criteria necessary for diagnosing NAFLD, MAFLD by definition, is closely associated with T2DM, obesity and atherogenic dyslipidaemia, which are established risk factors for CVD [27].

**Fig. 3:**
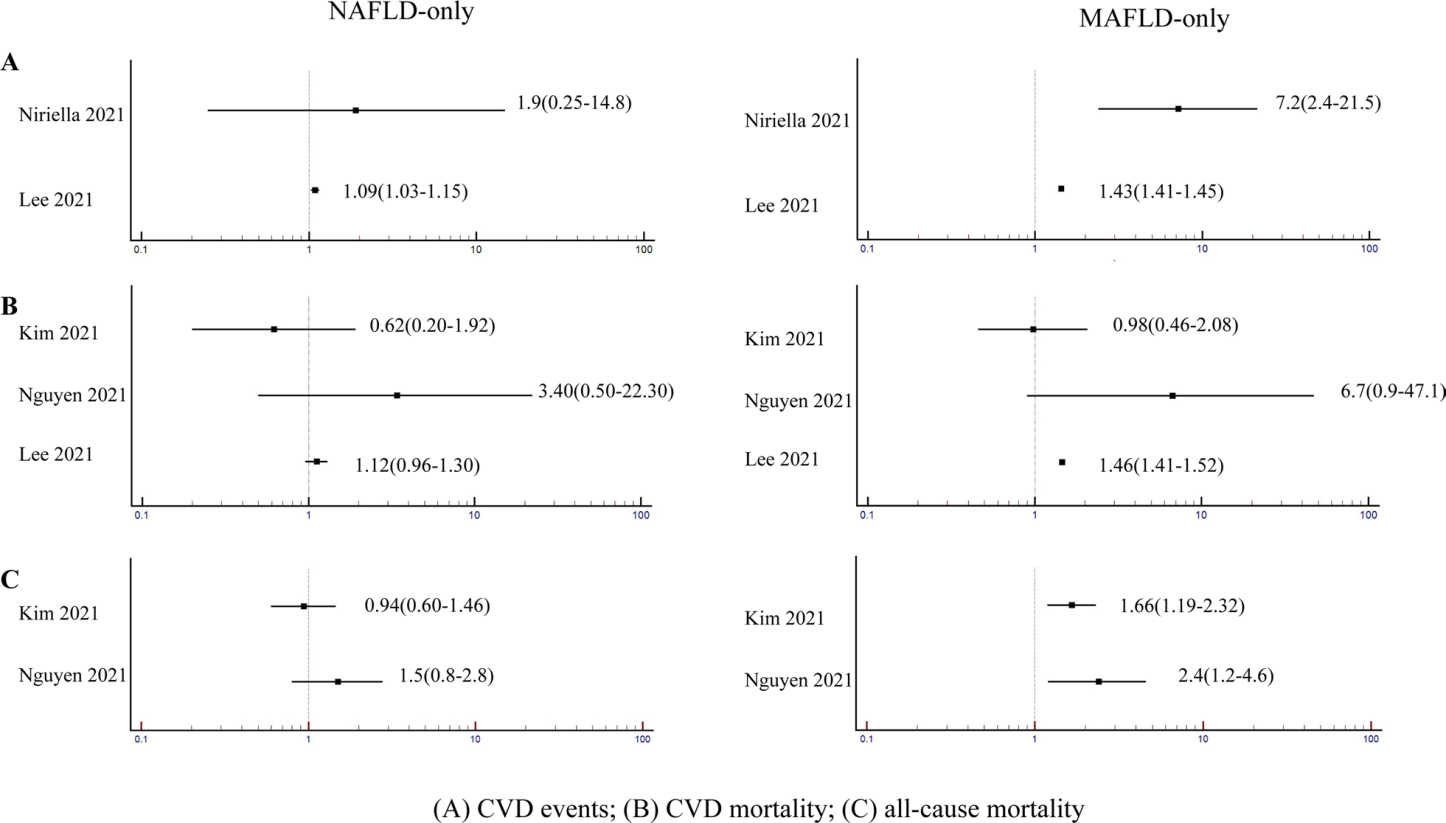
Comparative effects of MAFLD-only and NAFLD-only on the risk of fatal and nonfatal CVD events and all-cause mortality. The figure shows the forest plots of the effects of the MAFLD-only or the NAFLD-only status on the risk of CVD mortality and events and all-cause mortality in cohort studies that simultaneously used the MAFLD and NAFLD definitions. The NAFLD-only status is defined as presence of NAFLD but not MAFLD; the MAFLD-only status is defined as presence of MAFLD but not NAFLD. The reference category in these statistical analyses is the absence of both NAFLD and MAFLD. Data are expressed as hazard ratios and 95% confidence intervals (in parenthesis). (A) CVD events [14, 28]: the MAFLD-only status was associated with a higher risk of CVD events than the NAFLD-only status; (B) CVD mortality [14, 29, 30]: either the MAFLD-only status or NAFLD-only status was not associated with CVD mortality; (C) all-cause mortality [29, 30]: the MAFLD-only status was associated with a higher risk of all-cause mortality than the NAFLD-only status. Abbreviations: CVD: cardiovascular disease; MAFLD: metabolic dysfunction-associated fatty liver disease; NAFLD: non-alcoholic fatty liver disease

Recent cohort studies that compared MAFLD-only and NAFLD-only patient populations suggest that the MAFLD-only status is more strongly associated with risk of overall mortality, CVD mortality and nonfatal CVD events, compared with the NAFLD-only status (Fig. 3) [14, 28–31]. In particular, as shown in Fig. 3A, the MAFLD-only status seems to be more closely associated with a higher risk of nonfatal CVD events. In a retrospective cohort study of 2985 participants followed for 7 years, Niriella et al. [28] reported that the NAFLD-only status was not associated with CVD events compared with control individuals (HR = 1.90, 95% CI = 0.25–14.8) (although it should be noted the CIs were wide and the study may be underpowered), whilst the MAFLD-only status was associated with a greater risk of CVD events compared with control individuals (HR = 7.2, 95% CI = 2.4–21.5). In another study of ~ 9.5 million South Korean subjects from a health screening population, Lee et al. [14] reported that individuals with MAFLD only were at higher risk of CVD events compared with those without MAFLD or NAFLD (HR = 1.43, 95% CI = 1.41–1.45), whereas the association between the NAFLD-only status and risk of CVD events was modest (HR = 1.09, 95% CI = 1.03–1.15). As shown in Fig. 3B, in the study by Lee et al. [14], patients with MAFLD were also at higher risk of CVD mortality compared with individuals without MAFLD or NAFLD (HR = 1.46, 95% CI = 1.41–1.52), whereas NAFLD patients were not (HR = 1.12, 95% CI = 0.96–1.30). For all-cause mortality (Fig. 3C), the difference in CVD risk associated with MAFLD or NAFLD was even more apparent. Kim et al. [30] analyzed data from 7,761 participants in the NHANES-III database and showed that MAFLD was associated with a higher risk of all-cause mortality compared to those without MAFLD or NAFLD (HR = 1.66, 95% CI = 1.19–2.32), whereas NAFLD was not (HR = 0.94, 95% CI = 0.60–1.46). Similarly, Nguyen et al. [29] reported that the MAFLD-only status identified a group of patients with higher all-cause mortality compared with individuals without MAFLD or NAFLD (HR = 2.4, 95% CI = 1.2–4.6), whereas there was no increased risk for all-cause mortality with the NAFLD-only status (HR = 1.5, 95% CI = 0.8–2.8).

We recently performed a meta-analysis of seven observational cohort studies (mostly from Asian countries) that examined the comparative effects of NAFLD and MAFLD definitions on risk of CVD events [32]. This meta-analysis showed that each of the two definitions were significantly associated with a higher risk of incident CVD events (pooled random-effects HR 1.50, 95% CI 1.30–1.72 for MAFLD vs. no-MAFLD; and pooled random-effects HR 1.27, 95% CI 1.12–1.45 for NAFLD vs. no-NAFLD, respectively). Although MAFLD identified a numerically greater number of CVD events than NAFLD, the risk for incident CVD events associated with either definition was not significantly different [32].

Collectively, since the MAFLD definition better captures underlying metabolic dysfunction, it is perhaps not surprising that MAFLD definition might also increase CVD risk more strongly than NAFLD definition. However, further cohort studies from different countries are certainly needed to elucidate whether MAFLD may better predict the risk of developing incident CVD events than NAFLD.

## Why is MAFLD associated with an increased risk of CVD?

There are at least two possible explanations for the increased CVD risk observed in individuals with MAFLD. First, the MAFLD definition has as an obligate requirement for the presence of overweight/obesity, T2DM or other features of the metabolic syndrome, all of which are associated with increased CVD risk. In MAFLD, the presence of T2DM marks the most severe form of metabolic dysfunction and hence has the worst prognosis [33]. Indeed, recent studies have shown that MAFLD patients with T2DM have a worse clinical outcome than their counterparts without T2DM (i.e. MAFLD patients with overweight/obesity, or nondiabetic MAFLD patients with other metabolic risk abnormalities) [34]. Several pathophysiological pathways may link MAFLD and T2DM to an increased CVD risk, including a proatherogenic lipid phenotype, as well as an increase in prothrombotic factors, insulin resistance, low-grade inflammation, and intestinal dysbiosis [35].

Second, the impact of MAFLD on CVD risk may also be affected by other coexisting liver diseases, such as viral hepatitis or moderate alcohol consumption. Whereas it is necessary to always exclude these coexisting liver diseases to establish a diagnosis of NAFLD, this is not necessary for a diagnosis of MAFLD. Indeed, some studies showed that patients with MAFLD and concomitant viral hepatitis or moderate alcohol consumption have a higher 10-year calculated CVD risk compared to those with MAFLD only [29, 36, 37].

**Fig. 4:**
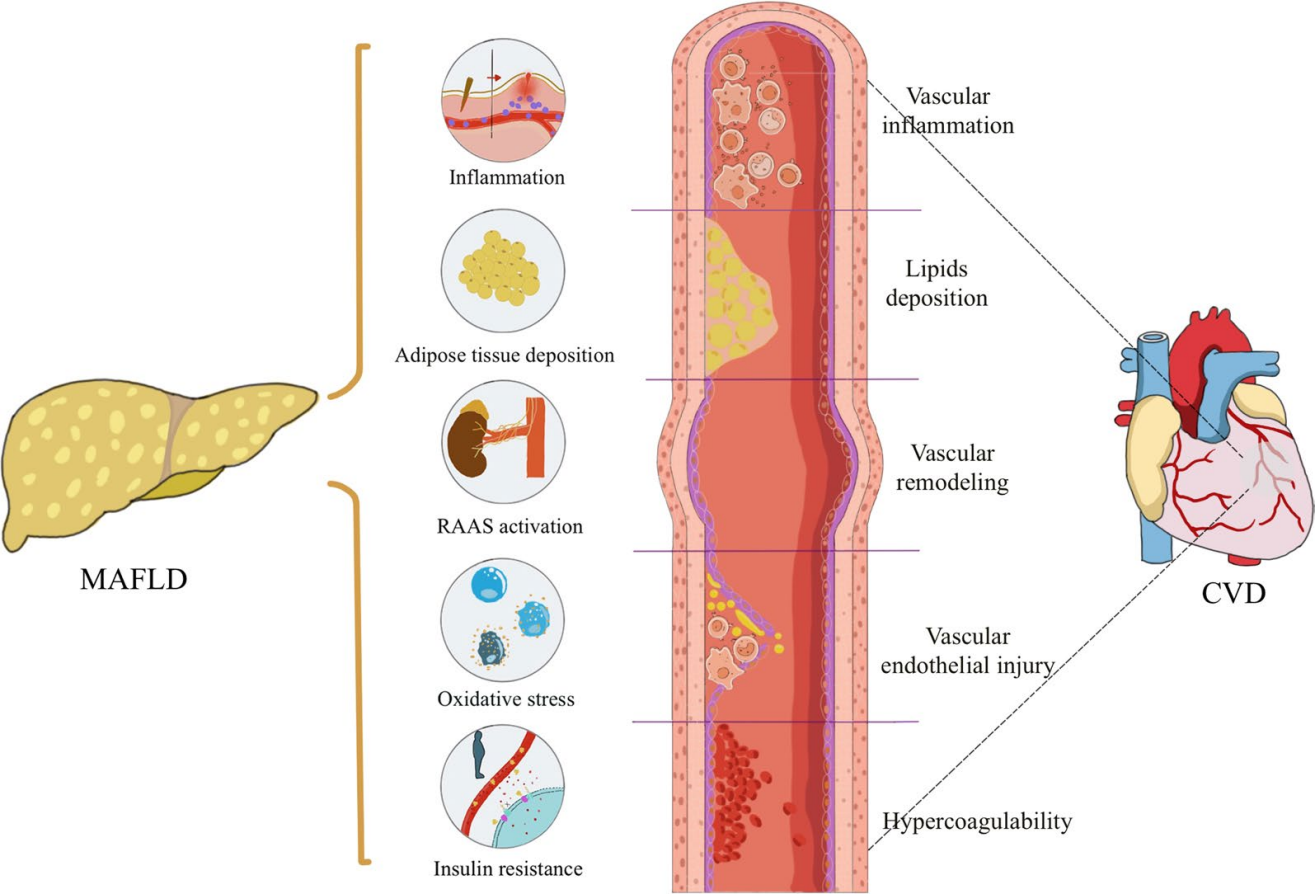
Putative shared pathophysiological mechanisms in MAFLD and CVD. MAFLD is closely associated with metabolic dysfunction and typical features of the metabolic syndrome. These metabolic risk abnormalities include visceral adipose tissue deposition, systemic low-grade inflammation, increased activity of RAAS systems, enhanced oxidative stress and insulin resistance. These metabolic risk abnormalities induce progression of coronary atherosclerosis, including vascular inflammation, lipids deposition, vascular remodeling, endothelial injury, as well as hypercoagulability, thereby contributing to increased risk of CVD. *CVD* cardiovascular disease, *MAFLD* metabolic dysfunction-associated fatty liver disease, *RAAS* renin–angiotensin–aldosterone system

That said, MAFLD itself may increase risk of CVD possibly via multiple pathophysiological mechanisms associated with metabolic dysfunction; these include increased oxidative stress, systemic/hepatic insulin resistance, low-grade inflammation and endothelial dysfunction (Fig. 4) [38–41]. Patients with MAFLD exhibit excessive reactive oxygen species (ROS), and ROS overproduction leads to hepatic inflammation and fibrosis, mostly through activation of hepatic stellate cells [42]. ROS overproduction also leads to low-density lipoprotein (LDL)-cholesterol oxidation, which may promote transformation of macrophages into foam cells, which is a key step in the formation of atherosclerotic lesions and atherosclerosis progression. The latter occurs through a variety of pathways, including endothelial cell dysfunction and vascular smooth muscle cell proliferation [43]. Insulin resistance is considered one of the core pathophysiological changes in MAFLD [44]. Insulin resistance promotes hepatic de novo lipogenesis and may affect microvascular and macrovascular homeostasis in a variety of ways to promote atherosclerosis [44]. In addition, previous studies confirmed that chronic hyperglycemia damages vascular endothelial cells, stimulates proliferation of smooth muscle cells, improves platelet activity, and induces ROS overproduction, thus promoting accelerated atherogenesis [45]. Low-grade inflammation also aggravates endothelial dysfunction, changes vascular tone, and promotes vascular plaque formation [46]. All these mechanisms promote the development and progression of CVD including vascular inflammation, lipid deposition, vascular remodeling, endothelial injury and hypercoagulability. Given that MAFLD is defined by the presence of hepatic steatosis *plus* at least one of its diagnostic cardiometabolic criteria [4], it is reasonable to hypothesize that there will be a strong mechanistic association between MAFLD and adverse CVD outcomes [18, 20, 47, 48].

## What is the role of MAFLD in CVD? Is it a simple bystander or a mediator?

**Fig. 5:**
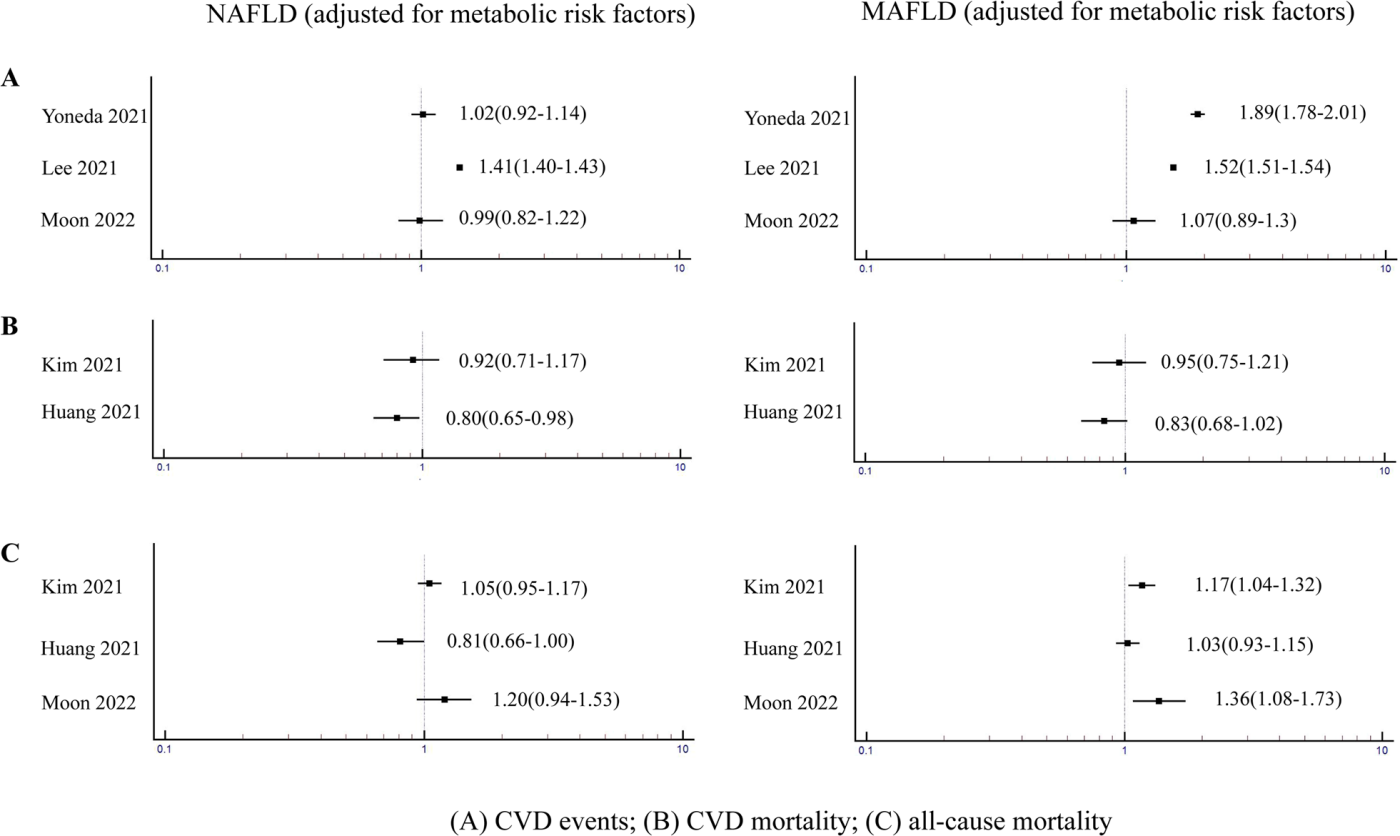
Comparative effects of MAFLD and NAFLD on the risk of fatal and nonfatal CVD and all-cause mortality independently of cardiometabolic risk factors. The figure shows the forest plots of the effects of MAFLD and NAFLD on the risk of CVD mortality and events and all-cause mortality after adjustment for coexisting cardiometabolic risk factors, in cohort studies that simultaneously used the MAFLD and NAFLD definitions. Data are expressed as hazard ratios and 95% confidence intervals (in parenthesis). **A** CVD events [14, 50, 52]: MAFLD is associated with a greater risk of CVD events than NAFLD **B** CVD mortality [30, 47]: the risk for CVD mortality is attenuated after adjustment for cardiometabolic risk factors in MAFLD or NAFLD; **C** all-cause mortality [30, 47, 50]: MAFLD is associated with a higher risk of all-cause mortality but this association is diminished after adjustment for cardiometabolic risk factors. *CVD* cardiovascular disease, *MAFLD* metabolic dysfunction-associated fatty liver disease, *NAFLD* non-alcoholic fatty liver disease

To date, most Cardiologists are not aware that NAFLD (or MAFLD) is a CVD risk factor [30, 49, 50]. In 2015, a position paper published by the Indian College of Cardiology [51] identified the increased CVD risk in patients with NAFLD, but raised some doubts as to whether NAFLD per se may predispose to CVD development. However, since the publication of that position paper, further new data has provided yet more evidence that MAFLD is a CVD risk factor [14, 52]. Interestingly, there is a discrepancy for the risk of CVD outcomes in MAFLD and NAFLD after adjustment for coexisting cardiometabolic risk factors (Fig. 5). As shown in Fig. 5A, in a cohort study of ~ 6.8 million Japanese individuals, Yoneda et al. [52] reported that the risk of CVD events was almost the same (adjusted HR 1.02, 95% CI 0.92–1.14) in the NAFLD and non-NAFLD groups after adjusting for cardiometabolic risk factors. In contrast, after adjusting for the same cardiometabolic risk factors, the risk of CVD was higher in the MAFLD group compared with the non-MAFLD group (adjusted HR 1.89, 95% CI 1.78–2.01). However, there are conflicting data (Fig. 5B) [38, 47, 53–55]. In a smaller prospective study, Kim et al. [30] reported a significant association between MAFLD and CVD mortality (HR 2.14, 95% CI 1.71–2.70), but this risk was attenuated after adjusting for cardiometabolic risk factors. Similarly, these authors did not find any association between NAFLD and CVD mortality in adjusted regression analyses. Using the NHANES III database, Huang et al. [47] reported that MAFLD was associated with a greater risk of CVD mortality compared with NAFLD (HR 2.01, 95% CI 1.66–2.44 vs. HR 1.53, 95% CI 1.26–1.86, respectively). However, the increased risk of CVD mortality was attenuated after adjustment for cardiometabolic risk factors. Previous meta-analyses reported that NAFLD was associated with a higher risk of nonfatal CVD events but not CVD mortality [55–57]. However, the largest updated meta-analysis to date by Mantovani et al. [39] has clearly shown that NAFLD was associated with a higher risk of both nonfatal CVD events (pooled random-effects HR1.40, 95% CI 1.20–1.64) and CVD mortality (pooled random-effects HR 1.30, 95% CI 1.08–1.56), and that this risk was further increased with the severity of NAFLD (especially with higher fibrosis stage). A nationwide Swedish cohort study by Simon et al. [58] provided further evidence of a strong association between the presence and severity of biopsy-proven NAFLD and the risk of CVD mortality.

MAFLD is associated with a higher risk of all-cause mortality but this association is attenuated after adjustment for cardiometabolic risk factors (Fig. 5C) [30, 47, 50]. For example, Kim et al. [30] reported that the association with higher all-cause mortality in MAFLD became non-significant, after adjustment for cardiometabolic risk factors. Huang et al. [47] showed that MAFLD was associated with higher all-cause mortality compared with NAFLD and control subjects, but the associations lost significance after adjustment for cardiometabolic risk factors, in both MAFLD and NAFLD. On the other hand, in a community-based cohort study of 8919 subjects Moon et al. [50] reported that MAFLD significantly predicted the risk of all-cause mortality even after adjustment for cardiometabolic risk factors (HR 1.36, 95% CI 1.08–1.73), whereas NAFLD did not (HR 1.20, 95% CI 0.94–1.53).

Although the recent AHA scientific statement identified NAFLD as an independent risk factor for CVD, the question as to whether MAFLD is a simple bystander or an active mediator in the pathogenesis of CVD remains [59]. Based on the available evidence [13], the shared cardiometabolic risk factors play an important role but likely do not account for the entire relationship between MAFLD and the risk of CVD events. Apart from shared cardiometabolic risk factors, the precise mechanism(s) underlying the association between MAFLD and CVD risk is (are) not clear, but some potential mechanisms (such as, for example, activation of the renin–angiotensin–aldosterone system, some NAFLD-related genetic polymorphisms and intestinal dysbiosis) may also play a role in both MAFLD and CVD [1], but further research is needed.

## Is routine screening for MAFLD necessary for CVD risk assessment?

Based on current evidence, whether a diagnosis of MAFLD improves CVD risk prediction remains uncertain [46, 60]. Currently, in high-risk patient populations with obesity, T2DM or MetS, screening for MAFLD has been recommended by many scientific guidelines [61–63]. Conversely, routine screening for MAFLD has not been recommended by scientific guidelines from cardiovascular societies [2, 13, 64]. Before MAFLD screening can be recommended, it is necessary to demonstrate that routine screening may improve both liver-related and cardiovascular outcomes in a cost-effective manner [65]. Wong et al. [66] performed a study of 612 patients referred for coronary angiography with 3679 patient-years of follow-up to test the utility of MAFLD for CVD risk prediction. These authors found that whilst the presence of MAFLD was associated with significant coronary artery disease and need for coronary revascularization procedures, the rates of mortality and CVD events were the same among the MAFLD and non-MAFLD patient cohorts.

In fatty liver disease, it is often overlooked that the severity of liver fibrosis is strongly associated with an increased risk of fatal and nonfatal CVD events [67]. Non-invasive tests for diagnosing liver fibrosis may reduce the number for unnecessary liver biopsies and identify patients at higher risk of CVD. As proof, in a population-based cohort study of 3512 individuals, Tamaki et al. [68] examined the associations between non-invasive biomarkers of liver fibrosis [including Fibrosis-4 (FIB-4) index, non-alcoholic fatty liver disease fibrosis score (NFS), and Wisteria floribunda agglutinin-positive Mac-2 binding protein (WFA^+^-M2BP)] and risk of CVD events. The authors showed that advanced fibrosis (defined as FIB-4 ≥ 2.67, NFS ≥ 0.675, or WFA^+^-M2BP ≥ 1.0) was associated with higher CVD risk (using the Framingham risk score), independent of traditional CVD risk factors. In another prospective study of nearly 900 outpatients with metabolic syndrome followed for a median period of 41 months, Baratta et al. [54] showed that subjects with NAFLD and FIB-4 ≥ 2.67 had a fourfold increase in fatal and nonfatal CVD events (HR 4.02, 95% CI 1.06–5.74). Although further prospective studies are needed, these findings are proof of concept for the use of non-invasive tools for a better CVD risk stratification in MAFLD.

## What is the effect of treatment interventions for MAFLD on the risk of CVD?

**Fig. 6:**
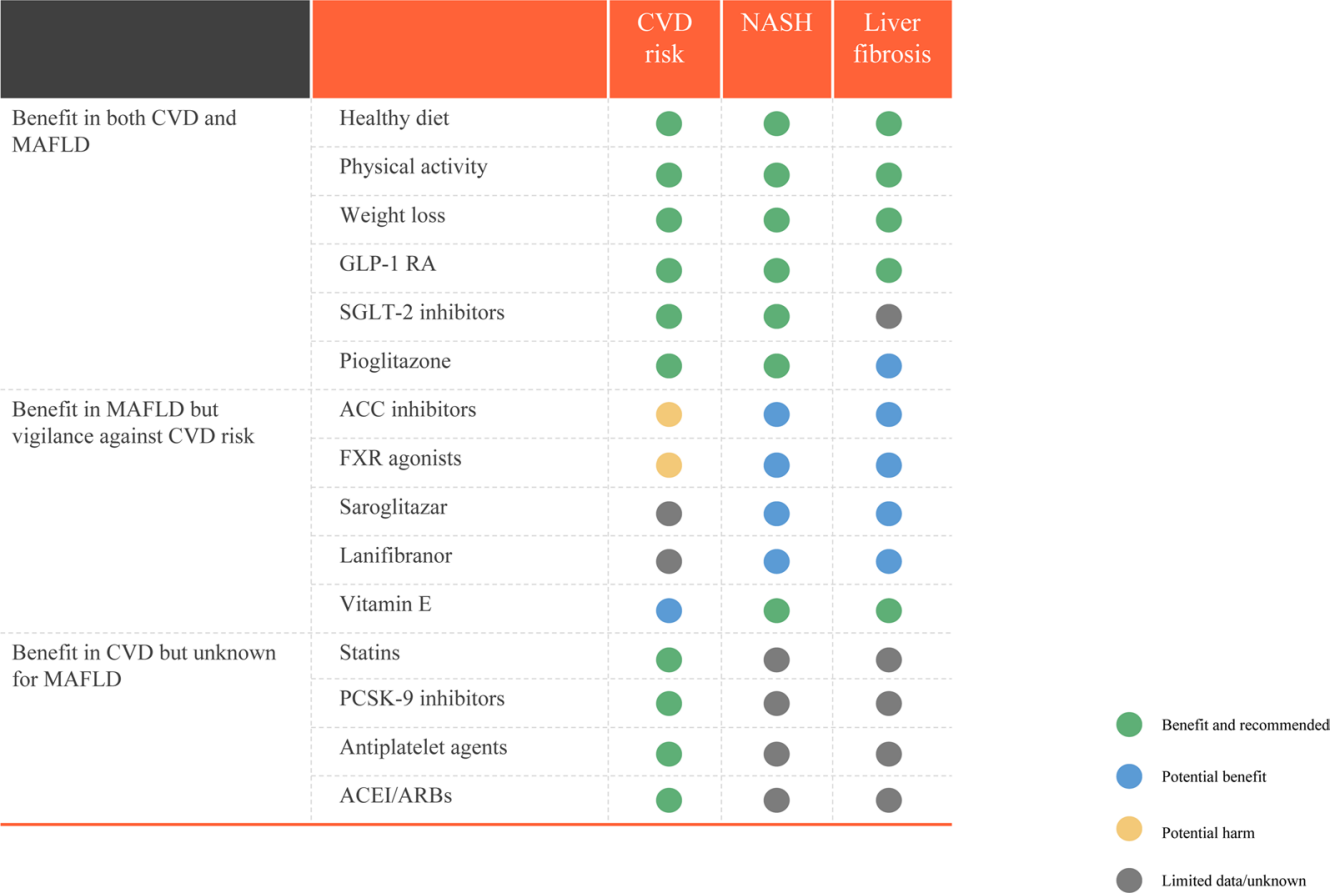
Assessment of lifestyle interventions and pharmacotherapies on CVD risk and liver histology features. *ACC* Acetyl-CoA carboxylase, *ACEi* angiotensin converting enzyme inhibitor, *ARBs* angiotensin II receptor blockers, *CVD* cardiovascular disease, *GLP-1RA* glucagon-like peptide 1 receptor agonist, *NASH* non-alcoholic steatohepatitis, *PCSK9* proprotein convertase subtilisin/kexin type 9, *SGLT-2i* sodium-glucose cotransporter 2 inhibitor

Safe, effective and acceptable pharmacotherapies for MAFLD must halt or delay the progression from simple steatosis to cirrhosis, end-stage liver disease and/or hepatocellular carcinoma. The efficacy and safety of potential treatments for MAFLD that reduce the risk of CVD are summarized in Fig. 6.

### Interventions with benefit in both CVD and MAFLD

Lifestyle intervention continues to play a key role in the primary and secondary prevention of CVD, as also recommended in several guidelines for management of MAFLD [63, 69, 70]. Adopting a diet rich in vegetables, fruits, legumes, nuts, whole grains and fish is recommended in order to reduce CVD risk and to improve hepatic steatosis and inflammation [64]. A Mediterranean-type diet may reduce hepatic steatosis, improve insulin resistance [71, 72], and is also effective in primary and secondary prevention of CVD [64, 73].

Weight loss is an essential treatment component for reducing CVD risk. Weight loss of 5% to 10% has been shown to be an achievable goal in most lifestyle interventions and results in significant improvements of hepatic histology features (steatosis, inflammation and fibrosis) and CVD risk reduction [63, 74]. With regard to physical activity, at least 150 min per week of accumulated moderate-intensity aerobic physical activity or 75 min per week of vigorous-intensity aerobic physical activity, can improve hepatic steatosis and reduce CVD risk [75, 76]. There may be no lower limit to the amount of moderate to vigorous physical activity at which the benefits of CVD risk reduction begins [77]. Therefore, for adults who cannot meet the minimum level of physical activity, engaging in some moderate or vigorous physical activity may help to reduce risk of CVD [76, 77].

Sleep is an emerging risk factor for cardiometabolic disease with strong relationships between obstructive sleep apnea and fatty liver disease, possibly mediated (at least in part) by recurrent nocturnal hypoxemia [78]. Importantly, in light of the increasing prevalence of inadequate sleep worldwide, sleep deprivation has been causally implicated in increased visceral fat deposition even in young and healthy subjects [79]. Indeed, the AHA recently included sleep in its list of “Life’s Essential 8”, as a behavioral strategy for improving cardiovascular and metabolic population health (see https://www.heart.org/en/healthy-living/healthy-lifestyle/lifes-essential-8).

T2DM is often present in MAFLD and diabetic cardiomyopathy is a risk factor for CVD [80]. Recent data also suggests that some newer glucose-lowering agents may not only improve the histological features of NAFLD, but also significantly reduce CVD outcomes because these agents induce weight loss and improve glycemic control [81–83]. Glucagon-like peptide 1 receptor agonists (GLP-1RAs) and sodium-glucose cotransporter 2 inhibitors (SGLT-2i) are two newer classes of glucose-lowering agents that are highly effective for both T2DM treatment and risk reduction of CVD and kidney outcomes [81, 84]. A meta-analysis of phase-2 randomized controlled trials demonstrated that treatment with GLP-1RAs (especially subcutaneous liraglutide and semaglutide) significantly reduce body weight and improve liver histology in NAFLD [85]. Tirzepatide, a novel, dual GLP-1RAs and glucose-dependent insulinotropic polypeptide (GIP) may also exert beneficial effects on liver fat content and the volume of visceral and abdominal subcutaneous adipose tissues. Importantly, tirzepatide did not increase the risk of major CVD events in patients with T2DM [86, 87]. Some phase-2 randomized controlled trials have reported that SGLT2i treatment may also improve hepatic fat content and fibrosis [88, 89]. Nevertheless, the beneficial effects of these newer glucose-lowering agents on hepatic fibrosis beyond weight loss require further study. Pioglitazone, a peroxisome proliferator-activated receptor (PPAR)-gamma agonist, is another glucose-lowering drug that also improves hepatic histology features in patients with biopsy-proven non-alcoholic steatohepatitis, irrespective of the coexistence of T2DM [82, 90]. The benefits of pioglitazone on CVD outcomes in patients with and without T2DM are also well-known [91, 92]. However, safety concerns and moderate weight gain have severely impacted the long-term use of this drug in clinical practice [93, 94].

### Therapies with benefit in MAFLD but with cardiovascular safety concerns

Acetyl-CoA carboxylase (ACC) is a key enzyme in fatty acid synthesis that has been explored as a therapeutic target for metabolic steatohepatitis [95]. ACC inhibitors may improve hepatic steatosis, inflammation and fibrosis [96]. Unfortunately, in a randomized controlled trial, ACC inhibitors reduced liver fat content but increased plasma triglyceride levels, raising concerns about their CVD safety [96]. To date, Mendelian randomization studies have not provided sufficient evidence to support the conclusion that hepatic fat accumulation is causally associated with CVD [97]. Conversely, some studies reported that MAFLD susceptibility genotypes (e.g., genetic variants in patatin-like phospholipase domain containing 3 (*PNPLA3*) and trans-membrane 6 superfamily member 2 (*TM6SF2*)) are associated with higher risk of fatty liver and steatohepatitis, but with a less atherogenic lipid profile and lower risk of CVD [98, 99].

Farnesoid X receptor (FXR) agonists have therapeutic potential for MAFLD by correcting abnormalities in intermediary metabolism and lipid accumulation, inhibiting p53 activation induced by metabolic stress, inhibiting the progression of fibrosis, and reducing hepatic inflammation [100, 101]. However, obeticholic acid as the first FXR agonist to be submitted for approval for treatment of nonalcoholic steatohepatitis was rejected by the U.S. Food and Drug Administration in 2020 citing uncertainty over the expected benefits based on alternative histopathological endpoints and after consideration that the treatment benefits did not outweigh the potential risks of increasing plasma LDL-C concentrations.

Saroglitazar, a peroxisome proliferator-activated receptor (PPAR) α/γ dual agonist is the first drug to be approved for non-cirrhotic non-alcoholic steatohepatitis (NASH). A randomized, double-blind, placebo-controlled trial demonstrated that high dose saroglitazar (4 mg daily) for 16 weeks reduced liver fat content and improved insulin resistance, serum triglyceride, and transaminase levels in obese patients with NAFLD or NASH [102]. Saroglitazar was approved in India in 2020, but regulatory approval outside of India has not occurred.

Lanifibranor is a pan-PPAR agonist that activates PPAR, α, γ and δ receptors. In the phase 2B placebo-controlled NATIVE trial [103], the histological SAF-A (activity of liver steatosis, activity, and fibrosis) score was reduced in obese patients with biopsy-confirmed nonalcoholic steatohepatitis. Additionally, multiple secondary endpoints were achieved with satisfactory resolution of steatohepatitis without worsening of fibrosis, and improvement in fibrosis stage of at least one stage without worsening of NASH. However, there is little evidence of its impact on CVD risk.

Vitamin E effectively improves hepatic histology in adult patients with biopsy-proven NASH [104]. Combined low-dose spironolactone plus vitamin E also decreased NAFLD liver fat score [105]. However, studies evaluating vitamin E for histological benefit have generally been negative or have produced inconsistent results in small groups of patients [106–108]. The results of some randomized placebo-controlled clinical trials also indicate that vitamin E supplementation not only failed to prevent major CVD events, but in fact may increase the risk of developing heart failure [109].

### Therapies with benefit in CVD but unknown or uncertain effects in MAFLD

Statins are the first-line treatment to prevent atherosclerotic CVD in patients with hypercholesterolemia [110]. Statins reduce the risk of CVD in MAFLD patients with dyslipidemia, even without any beneficial effect on liver histology [63, 111]. Statins are known to be safe in NAFLD and statin use is not associated with abnormal serum liver enzyme levels, even in patients with hepatic steatosis [112–114]. An unexpected concern is that statin treatment might be suboptimal for subjects with MAFLD [114], however further research is needed to test this further. Proprotein convertase subtilisin/kexin type 9 (PCSK9) inhibitors represent an alternative pharmacological approach to reducing plasma LDL-C concentrations. While some studies reported a possible beneficial effect on hepatic pathology, it is premature to recommend this agent for specifically treating MAFLD.[115–117] Daily aspirin use has been associated with fewer severe histologic features of MAFLD and a lower risk of progressing to advanced fibrosis in a recent observational study [118]. Angiotensin converting enzyme inhibitors (ACEi) and angiotensin II receptor blockers (ARBs) are also thought to exert a moderate anti-fibrotic effect on the liver in experimental and clinical studies [119]. Given the current evidence, more and larger controlled clinical trials are needed before a recommendation for use of these anti-hypertensive agents can be recommended for specifically treating MAFLD.

## Conclusions

The AHA statement in 2022 [13] identifies liver fat accumulation (NAFLD) as an independent risk factor for CVD. However, routine screening for MAFLD in patients with pre-existing CVD is not currently recommended. There is increasing scientific and clinical interest in the link between MAFLD and CVD risk, not least because newer glucose-lowering drugs, such as GLP-1RAs and SGLT2i may exert benefit on both hepatic fat content and CVD outcomes. That said, when safe and effective pharmacological treatments for MAFLD are licensed, management will involve close liaison between Cardiologists and other physicians treating this multisystem disease.

## Data Availability

Not applicable.

## Abbreviations

ACC: Acetyl-CoA carboxylase
ACEi: Angiotensin converting enzyme inhibitor
ACS: Acute coronary syndrome
AHA: American heart association
ARBs: Angiotensin II receptor blockers
CVD: Cardiovascular disease
FDA: Food and drug administration
FIB-4: Fibrosis-4 index
FXR: Farnesoid X receptor
GLP-1RA: Glucagon-like peptide 1 receptor agonist
LDL-C: Low-density lipoprotein cholesterol
MAFLD: Metabolic dysfunction-associated fatty liver disease
MetS: Metabolic syndrome
NAFLD: Non-alcoholic fatty liver disease
NFS: Non-alcoholic fatty liver disease fibrosis score
NASH: Non-alcoholic steatohepatitis
NHANES III: Third National Health and Nutrition Examination Survey
OR: Odds ratio
PCSK9: Proprotein convertase subtilisin/kexin type 9
SGLT-2i: Sodium-glucose cotransporter 2 inhibitor
PPAR: Peroxisome proliferator-activated receptor
ROS: Reactive oxygen species
T2DM: Type 2 diabetes mellitus
WFA^+^-M2BP: Wisteria floribunda agglutinin-positive Mac-2 binding protein
